# The promastigote surface antigen gene family of the *Leishmania *parasite: differential evolution by positive selection and recombination

**DOI:** 10.1186/1471-2148-8-292

**Published:** 2008-10-24

**Authors:** Alain Devault, Anne-Laure Bañuls

**Affiliations:** 1 Génétique et Evolution des Maladies Infectieuses, IRD/CNRS (UMR 2724), Montpellier F-34394, France; 2 CRBM-CNRS (UMR5237), Montpellier F-34293, France

## Abstract

**Background:**

PSA (promastigote surface antigen) is one of the major classes of membrane proteins present at the surface of the parasitic protozoan *Leishmania*. While it harbours leucine rich repeats, which are suggestive of its involvement in parasite-to-host physical interactions, its exact role is largely unknown. Furthermore, the extent of diversity of this gene family, both in copy number and sequence has not been established.

**Results:**

From the newly available complete genome sequences of *L. major*, *L. infantum *and *L. braziliensis*, we have established the complete list of *PSA *genes, based on the conservation of specific domain architecture. The latter includes an array of leucine rich repeats of unique signature flanked by conserved cysteine-rich domains. All *PSA *genes code either for secreted or membrane-anchored surface proteins. Besides the few previously identified *PSA *genes, which are shown here to be part of a relatively large subclass of *PSA *genes located on chromosome 12, this study identifies seven other *PSA *subtypes. The latter, whose genes lie on chromosomes 5, 9, 21 and 31 in all three species, form single gene (two genes in one instance) subfamilies, which phylogenetically cluster as highly related orthologs. On the other hand, genes found on chromosome 12 generally show high diversification, as reflected in greater sequence divergence between species, and in an extended set of divergent paralogs. Moreover, we show that the latter genes are submitted to strong positive selection. We also provide evidence that evolution of these genes is driven by intra- and intergenic recombination, thereby modulating the number of LRRs in protein and generating chimeric genes.

**Conclusion:**

PSA is a *Leishmania *family of membrane-bound or secreted proteins, whose main signature consists in a specific LRR sequence. All *PSA *genes found in the genomes of three sequenced *Leishmania *species unambiguously distribute into eight subfamilies of orthologs. Seven of these are evolving relatively slowly and could correspond to basic functions related to parasite/host interactions. On the opposite, the other *PSA *gene class, which include all so far experimentally studied *PSA *genes, could be involved in more specialised adaptative functions.

## Background

*Leishmania *is a parasitic protozoan of the Trypanosomatidae family and is the pathogenic agent of leishmaniases in humans [1,2]. These diseases are endemic in 88 countries on five continents, and cover a wide range of symptoms, from asymptomatic form and benign localised cutaneous lesions to mucocutaneous lesions and visceral outcome. While localised cutaneous lesions can be self-healing, the outcome of the other forms of the disease is more disabling, and the visceral forms are generally fatal if untreated by chemotherapy. The nature of these symptoms appears statistically associated with the parasite species. *L. major *and *L. tropica *mainly produce benign cutaneous forms, *L. braziliensis*, mucocutaneous forms and *L. infantum *and *L.donovani *generally cause visceral disorders. *Leishmania *undergoes two major developmental stages in its life cycle: one stage in an invertebrate host, the phlebotomine sand fly and one stage in a vertebrate host (wide range of hosts such as humans, canids, rodents, etc.). In the former, it proliferates in the sandfly gut as a flagellated form, promastigote, and later differentiates into the infectious metacyclic promastigote form while in vertebrate hosts it proliferates intracellularly as an a-flagellated form, amastigotes, after infection of macrophages. The parasite transits from one host to the other when the sandfly feeds by sucking the blood of the vertebrate host.

To understand host-parasite interactions at the molecular level, many studies have focused on the potential role of macromolecules present at the surface of the parasite. These include glycoinositolphospholipids (GIPL), lipophosphoglycan (LPG), as well as the membrane proteins proteophosphoglycan (PPG), MSP/GP63 endopeptidases, PSA (PSA-2/GP46) and amastins. Most of these membrane proteins are glycosylated and anchored via a covalent glycosylphosphatidylinositol (GPI) moiety [3]. Some isoforms of PPG and PSA are also exported outside the parasite as soluble proteins [4,5]. LPG plays an important role in binding to the phlebotomine midgut. Furthermore, in conjunction with the MSP proteins, it is involved in the first steps of the vertebrate host infection. They promote resistance to complement, binding to and internalisation in macrophages through the CR3 ligand and fibronectin receptors, and finally inhibition of the oxidative burst [6,7]. GIPLs and the cysteine proteases CPB are involved in later stages of infection like protection against the nitric oxide production and modulation of the immune response [8].

PSA proteins have been detected in both promastigote and amastigote stages [9,10]. However, the levels drastically increase from exponentially growing to stationary phase parasites, which suggests that PSA are strongly overexpressed in metacyclic promastigotes. This overexpression seems linked with the virulence status of the parasites since serial passaged *in vitro *cultured promastigotes with low virulence do not show this burst of PSA expression in stationary phase [10]. This expression profile is consistent with the only known role for PSA: resistance to complement lysis [11]. But other roles are possible, either in the late parasite-phlebotomine or metacyclic/amastigote-macrophage interactions. The most suggestive functional determinant in the PSA2 primary structure is the presence of leucine rich repeats (LRR). LRRs are primarily known to be involved in protein-protein and protein-glycolipids interactions. Interestingly, many known LRR containing proteins are involved in host-pathogen interactions. *Yersinia *invasin, *Listeria *internalins and *Streptococci *LrrG protein are surface proteins that play key roles in binding and internalisation of these bacteria in their mammalian hosts [12,13]. Likewise, the mammalian Toll-like receptor and Nod families [14], and the plant NBS-LRR resistance proteins [15] are primary actors of the innate immunological system that recognize and bind proteins, glycolipids and nucleic acids of pathogens.

As for the other membrane proteins above, PSAs are encoded by multicopy genes. Up to now, only one or two *PSA *genes have been described in *L. amazonensis *[16], *L. major *[9] and *L. infantum *[17], and before the complete sequencing of *L. major *genome, the number of copies in a single species was largely unknown. Here we have taken advantage of the newly available complete genome sequences of the three species *L. major*, *L. infantum *and *L. braziliensis *[18,19] to draw the precise structure of the *PSA *gene family. After analysis of the gene phylogeny, which might serve in the identification of distinct PSA functions, we have investigated for the presence of recombination and positive selection as driving forces acting on the evolution of this multigene family.

## Results

### The *PSA *gene family

**Figure 1:**
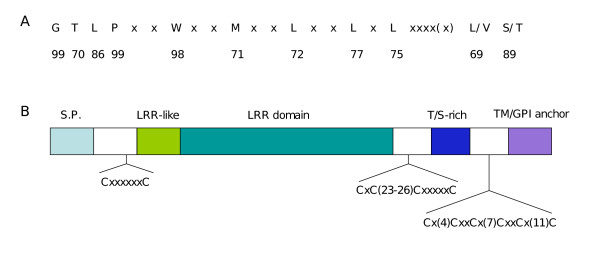
**Analysis of the primary structure of PSA proteins**. A) Consensus sequence of the PSA LRR repeats. Numbers indicate the percentage of occurrence in the LRR repeats of the corresponding residues above. Residues in position #3, 7, 10, 13, 16, 18, 23(or 24) are always hydrophobic. B) Schematic representation of the PSA domain architecture. S.P: signal peptide; T/S: threonine/serine TM: transmembrane domain.

**Figure 2:**
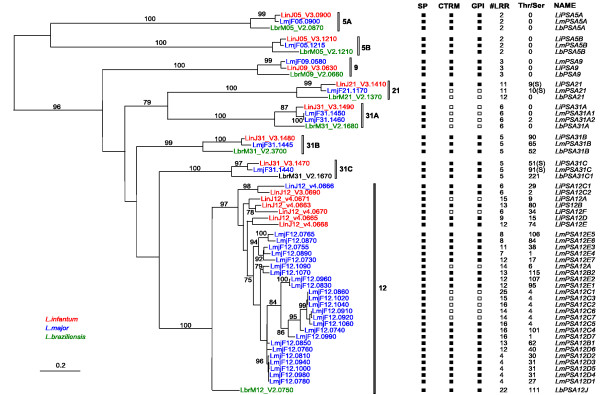
**Phylogenetic analysis of the *PSA *gene family**. A phylogenetic tree was inferred by PhyML for a DNA alignment of the *PSA *ORFs of *L. braziliensis*, *L. major *and *L. infantum*, excluding the Thr/Ser and Cys-rich/transmembrane C-terminal domains as well as all but the C-ter most LRR repeat (see Additional file 1). Only bootstrap values (from 500 replicates) greater than 74% are indicated. Very similar trees (with same significant clustering of genes) were obtained with DNA or protein sequence alignments, using both maximum likelihood or distance methods (not shown). SP: signal peptide; #LRR: number of LRR repeats (number in brackets refers to additional LRRs that diverge from the consensus LRR sequence). T/S: number of Thr and Ser residues in the Thr-rich domain (or Ser-rich, indicated by (S)). CRTM: cysteine-rich and C-ter transmembrane domains. GPI: GPI anchor. Filled and open squares indicate the presence or absence of the above features, respectively.

A BLAST search analysis was performed against the complete genome sequences of the three species *L. major*, *L. infantum *and *L. braziliensis *http://www.genedb.org/, using as a query sequence some of the PSA protein sequences reported in the literature. Some genes were discarded from the candidate *PSA *genes obtained from this BLAST analysis: LmjF12.1005, LinJ12_v4.0662, LbrM_V2.1660 and proteophosphoglycan (PPG) genes (4 in each species). LmjF12.1005 of *L. major *and LinJ12_v4.0662 of *L. infantum*, which code for N-terminal truncated forms of PSA (LmjF12.1005 protein actually lacks a LRR domain) are probably pseudogenes. LbrM_V2.1660 is almost identical to gene LbrM_V2.1670, besides harbouring a N-terminal truncation, but this is questionable since the genomic sequence immediately upstream LbrM_V2.1660 is lacking, which renders the determination of the N-terminus uncertain. PPGs indeed possess PSA-related LRR motifs, but their overall domain architecture differs from that of PSA proteins. More importantly, PPGs are most probably functionally different from PSA, owing to their very long (often more than 1000 residues) serine-rich stretches on which are anchored very large amounts of phosphoglycan chains [5]. At the end, 32, 14 and 8 *PSA *genes were found in *L. major*, *L. infantum *and *L. braziliensis*, respectively. Analysis of the primary structure of the encoded PSA proteins reveals that they all possess the same architecture (Fig. 1). The central part of the protein is composed of LRRs in tandem. These repeats, which show high variability, both in terms of number of repeats and primary sequence, are flanked by relatively well conserved domains, including Cys-rich domains, which are expected for extracellular LRR-containing proteins [20]. On the N-terminal side of the LRR domain, one finds a 29 to 31 residue-long domain reminiscent of the LRR motif, but which is clearly not a LRR. This is preceded by a Cx(6)C domain and finally by a signal peptide. At the other extremity, the last LRR is followed by a CGCx(23–26)CxxxxxC containing domain. This marks the end of the sequence for some PSA proteins. Others have extensions that contain either a Thr/Ser-rich region of variable length or a transmembrane domain preceded by a Cys-rich region, or both. This configuration of a N-terminal signal sequence and a C-terminal transmembrane domain predicts that all PSA proteins are either secreted or anchored to the plasma membrane by their C-terminal end. Furthermore, all PSA proteins containing a C-terminal transmembrane domain (and a signal peptide) are predicted to be GPI-anchored (see Fig. 2).

The main signature of PSA proteins is their specific 24–25 amino-acids LRR motif, of consensus sequence G(T/S)LPxxWxx (M/I)xxLxxLxLxxxx(x)(V/L/I)(S/T) (Fig. 1). The most striking feature of this motif relative to other LRRs is the presence of quasi invariant glycine, proline and tryptophane residues at positions 1, 4 and 7, respectively. This motif is not present in the predicted proteins from the fully sequenced genomes of two other trypanosomatidae, *T. brucei *and *T. cruzi*, and no clear homologs of *PSA *could be identified in these organisms. The only PSA domain for which homology was found in the other trypanosomatidae is the Thr/Ser-rich domain. The T(n)KP2 and T(n)EAPT repeats found in most of these domains are present in *T. cruzi *mucins. However, while it makes up the bulk of the mucin protein architecture, this domain is not always present in PSA proteins (see Fig. 2), and mucins do not contain any LRR repeat. Therefore, PSA proteins and mucins are likely to play different roles. Actually, the LRRs mostly similar to those of *Leishmania *PSA proteins belong to the plant NBS-LRR resistance proteins.

*PSA *genes are dispersed on chromosomes (Chr) 5, 9, 12, 21 and 31 for all three *Leishmania *species. Chromosomes 9 and 21 contain a single gene, while Chr 5 contains 2 genes, and Chr 31, 3 genes (4 in *L. major*). *PSA *genes localised on different chromosomes (and also those of the different Chr31 loci) are sufficiently divergent to define *PSA *gene classes (see below). Inside each chromosome, all *PSA *genes are clustered in tandem, except those of Chr 5 and gene LbrM31_V2.3700, which are well separated. The *PSA *subfamily present on Chr 12 is the most complex (except in *L. braziliensis *where there is only one gene). In *L. major*, the 24 Chr 12 *PSA *genes belong to a single cluster of tandem repeats, with many of these genes separated by a single non-related intervening gene. This pattern is also observed for the 7 Chr12 *PSA *genes of *L. infantum*. All *PSA *genes arranged in clusters have the same orientation relative to transcription. The only *PSA *genes studied so far *in vivo *belong to the Chr12 array. Although the *PSA *genes we have identified on the other chromosomes show significant sequence divergence with the Chr12 genes (see below), the conservation of the precise domain organisation between all these genes justifies their being included in the same unique *PSA *family.

### Phylogeny of the *PSA *gene family

An alignment of all PSA protein sequences from all three *Leishmania *species was generated. For phylogenic analysis, only the domains present in all PSA proteins were included in the alignment. The Thr/Ser and Cys-rich/transmembrane C-terminal domains were thus discarded, as well as the central LRR domain (except for the last LRR motif of each sequence, which was retained). LRR units probably evolve not only by nucleotide substitution but also by birth and loss of repeats following recombination events. Therefore, alignments of rather distant LRR domains (with low similarity between repeats and varying number of repeats) may not reflect properly their evolution. This truncated alignment was transformed into its DNA coding version (Additional file 1), which was used to generate a phylogenetic tree by PhyML (Fig. 2). This tree shows the existence of eight *PSA *subfamilies which can be defined by their chromosome localisation: Chr5 (A and B), 9, 12, 21 and three subfamilies on Chr31 (A, B, C). Subfamilies Chr5A and B are the most distantly related and pair apart from all other clusters. Among the latter, only the Chr21/Chr31A subfamilies can be phylogenetically linked with high confidence bootstrap values. Each of the three *Leishmania *species has at least one gene in each of these eight subfamilies. The *L. infantum *gene orthologs always group with those of *L. major*, which is expected from the known species tree. The relatively short branch length separating orthologs in comparison to those separating the inparalogs (ancestors of the seven gene subfamilies) might suggest that speciation of the three *Leishmania *is a late event in the evolution history of the PSA genes. In keeping with this interpretation, a tree with similar relative branch lengths was inferred when using the rate of synonymous substitutions (*d*_S_), a more neutral distance metric (not shown).

### Chr5A, B, 9, 21, 31A, B, C subfamilies of *PSA *genes

While the Chr12 subfamily is most diversified, having very different numbers (1 to 24) of rather distantly related paralogs in the three species, the *PSA *genes lying on the other chromosomes form very simple clusters. For each of the latter subfamilies, each species contains a single gene (except *L. major*, which has two highly similar paralogs for the Chr31A cluster) and the orthologs or pseudo-orthologs are quite similar (median = 75% amino acid identity for all three species and 93% for *L. infantum*/*L. major *orthologs). This degree of homology between orthologs, which was calculated on the same domains used above for phylogenetic tree determination, was also observed over the entire LRR domain. Indeed, confident alignments covering this domain can be obtained inside each of the Chr5A and B, 9, 21, 31A, B, and C clusters since the number of LRRs differ at most by one inside each subfamily and the similarity between orthologs is (median = 80% amino acid identity for all three species and 96% for *L. infantum*/*L. major *orthologs) (see Additional files 2, 3, 4, 5, 6, 7, 8 for sequence alignments). On the other hand, similarity between genes of different subfamilies is rather low over this domain (median = 32% amino acid identity, and much difference in number of repeats). This may suggest that the LRR domains characteristic of each cluster represent specific binding properties, which could confer specialized roles to their respective PSA proteins. Another link between these subfamilies and functional specialisation is given by the nature of the C-terminal domain. Indeed, all members of the same subfamily share the same membrane anchorage/secretion determinant. In particular, all genes of the phylogenetically linked Chr21 and 31A subgroups are predicted to code for secreted proteins (Fig. 2).

### Chr12 subfamily of *PSA *genes

**Figure 3:**
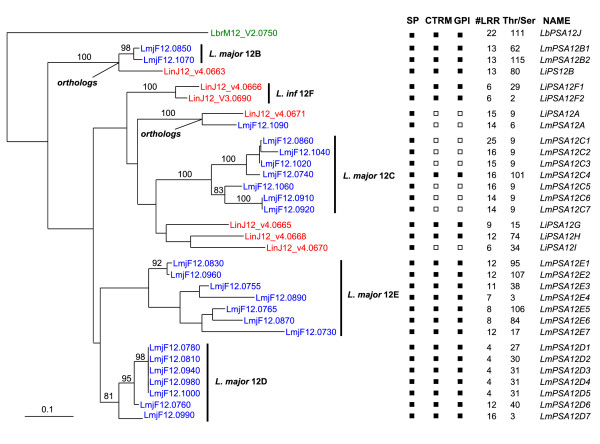
**Phylogenetic tree of the LRR domains of *PSA *genes located on chromosome 12**. A phylogenetic tree was inferred by PhyML for a DNA alignment of the LRR domain (see Additional files 9 and 13 for protein and DNA alignments, respectively) of all Chr 12 *PSA *genes of *L. braziliensis*, *L.major *and *L. infantum*. Bootstrap values (from 500 replicates) greater than 74% are indicated. Very similar trees (with same significant clustering of genes) were obtained with DNA or protein sequence alignments and by maximum likelihood or distance methods (not shown). SP: signal peptide; #LRR: number of LRR repeats. T/S: number of Thr and Ser residues in the Thr-rich domain (or Ser-rich, indicated by (S)). CRTM: cysteine-rich and C-ter transmembrane domains. GPI: GPI anchor. Filled and open squares indicate the presence or absence of the above features, respectively.

The tree of Fig. 2 shows that the phylogenetic structure of the Chr12 genes is quite different from that of the above subfamilies. Here, all genes group according to the species, which suggests that generation of paralogs in *L. major *and *L. infantum *occurred independently in each species. This clustering pattern was also obtained for an alignment corresponding to the C-ter extension containing the Cys-rich/transmembrane domains of the relevant subset of Chr12 *PSA *genes (not shown). We then produced an alignment of the LRR domain for these Chr12 genes. Although sequence diversity and variability in numbers of repeats render this task uncertain, this alignment could be validated by the high similarity between many genes and singularities of some LRRs (length of LRRs, sequence specificities). The most robust portion of this LRR alignment (Additional file 9) was used to generate a phylogenetic tree (Fig. 3). While many clades were conserved from the tree of Fig. 2, new well supported clusters were generated. Notably, two link together genes from *L. major *and *L. infantum*, thus identifying clear orthologs: LmjF12.1090/LinJ12_v4.0671 and LmjF12.0850, LmjF12.1070/LinJ12_v4.0663. Phylogenetic analysis scanning of all individual domains including single LRR repeats confirmed the above orthologies for all LRR repeats except those at both ends of the LRR domain (not shown). On the other hand, the N- and C-terminal domains, particularly the signal peptide region and the Cys-rich/transmembrane domain are species specific. The identification of LmjF12.1090/LinJ12_v4.0671 as orthologous genes was also strengthened by the absence of C-terminal Cys-rich and transmembrane coding domains in their respective ORFs. Yet, these domains are still encoded by very similar sequences located immediately downstream of the stop codons, suggesting that a common frameshift mutation resulted in this deletion. Besides the above orthologs, some clustering of *L. infantum *with *L. major *genes was observed, but with low bootstrap values. New trees were generated for the *L. infantum *genes alone, or for those of *L. major*, but they did not reveal any new well-supported clusters. If we consider the LRR domain as the main functional determinant of the PSA protein, the following picture of the genes on Chr12 emerges. In *L. infantum*, only two genes (LinJ12_v4.0666 and LinJ12_V3.0690) are closely related. Besides this clade, the history of the *L. infantum *Chr12 genes can not be resolved, due to the important apparent divergence between the sequences, which might result in part of recombination events involving LRR repeats. In *L. major*, we can distinguish four clusters of paralogs. Only three of them are supported by strong bootstrap values. Cluster Lm12B contains the two pseudo-orthologs of LinJ12_v4.0663. Subgroup C comprises seven genes, out of which six code for secreted proteins. As for the LmjF12.1090/LinJ12_v4.0671 genes, truncation of the Cys-rich and transmembrane domains in these genes originated from a common non-sense frameshift mutation. Cluster D comprises seven genes, five of which are almost identical (>99% nucleotide identity) and were most probably created by recent duplication events. In comparison to *L. infantum*, *L. major *has a much younger cluster of Chr12 genes, with 9 recently duplicated genes (*d*_S _<0.02 and no insertions or deletions). We must note, however, that nearly identical copies of *PSA *genes might have been missed in the *L. infantum *genome assembly, where contigs were assembled solely from shotgun sequence reads.

### Recombination in *PSA *genes

**Figure 4:**
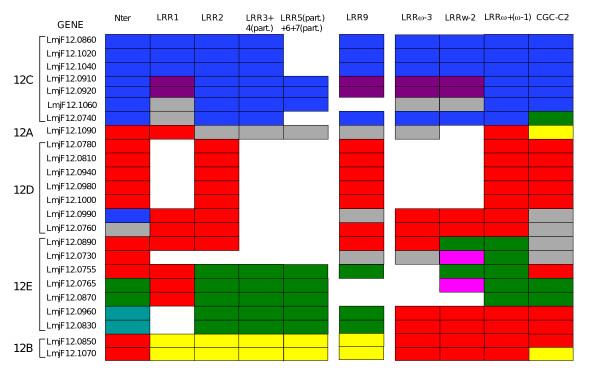
**Phylogenetic clustering of individual PSA domains**. Trees were inferred by Neighbor-Joining (NJ) from nucleotide distance matrix (TN93 model) calculated individually for all the indicated domains of the *L. major *Chr12 *PSA *genes. Individual LRR domains that were present in only a few genes were not analysed. For each domain, those genes that cluster together in the tree (with bootstrap values greater than 74%) are boxed with the same colour. Grey boxes indicate genes that do not cluster with any other genes. Absence of a box indicates that the corresponding domain is absent in the gene. N-ter: PSA N-terminal domain up to the first LRR repeat. LRRs are numbered from N-ter to C-ter according to the LRR alignment (see Additional file 9). *ω *: refers to the last (C-ter most) LRR of each gene; therefore, LRRω + (ω-1) corresponds to the sequence comprising the last and penultimate LRRs in each gene. (part.): partial; for example, 4 (part.) means portion of LRR 4. CGC-C2: the CGCx(23–26)CxxxxxC domain at the C-ter end of the LRR domain.

**Figure 5:**
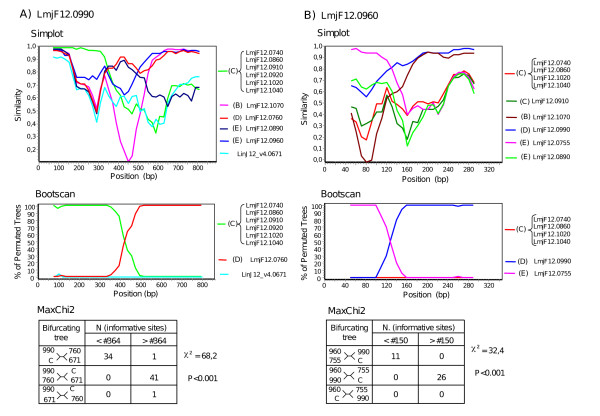
**Analysis of chimeric *PSA *genes by Simplot**. A) LmjF12.0990. A DNA alignment of the indicated genes covering LRR #2, 9 and the last 4 C-ter most LRRs, together with the N-ter and CGC-C2 flanking domains (Additional file 14) was analysed with Simplot. Single or multiple genes from each phylogenetic *PSA *gene cluster of *L. major *Chr 12 (see Fig. 3) were first used for similarity analysis (Simplot) against the LmjF12.0990 putative chimeric gene. The six genes of cluster 12C were reduced to a consensus sequence. The best pair of candidate parental genes (LmjF12.0760 and consensus sequence for cluster 12C) and a control gene were then used for bootscanning and informative sites analysis (MaxChi2). B) LmjF12.0960. The same analysis was performed as in A) using a DNA alignment covering LRR #2, 9 and the last 3 C-ter most LRRs (see Additional file 15).

The repetitive nature of the LRR domain and the number of paralogs imply that recombination has largely contributed to the diversity of the PSA gene. Some recombination events can be traced in the LRR domains. For example, while LRRs inside a given PSA protein are generally quite divergent, nearly identical repeats can be found. For example, LbrM12_V2.0750 has tandem triplicates of a LRR doublet, and LmjF12.0860, 1020, 1040 have up to 19 copies of a conserved LRR motif. These nearly exact duplications could have been generated by recent intragenic cross-overs, or cross-overs involving closely related paralogs. More diverging chimeric genes could result from intergenic recombination. To detect such events, we partitioned the LRR alignment of the *L. major *Chr12 genes into single LRR alignments and inferred the phylogenetic tree for the most conserved of them. Fig. 4 shows how these genes cluster according to these LRRs as well as to the conserved regions outside the LRR domain. It can be noticed that while genes from clade C are quite homogeneous in their clustering pattern across the entire alignment, others are more mosaic. This is the case for most of the genes of clade E, and this probably explains the low resolution (weak bootstrap values) of this subtree (see Fig. 3). Simple intergenic recombination events are readily suggested. For example, genes LmjF12.0830 and 12.0960 appear to be chimeric products originating from strand exchanges between genes belonging to clades D and E. Likewise, gene LmjF12.0990 must have been generated by the fusion of the N-terminal pre-LRR domain of a gene from clade C with a gene of clade D. To validate these conclusions, alignments of relevant *PSA *gene domains were analysed by SimPlot (Fig. 5) [21]. First, similarity profiles against the putative chimeric genes (LmjF12.0830, LmjF12.0960 and LmjF12.0990) show a clear switch between potential parent genes at the breakpoint region suggested above in Fig. 4. Second, bootscanning analysis reveals an alternation of high bootstrap support across the same regions. Finally, these recombination events are confirmed by analysis of informative sites by the Maximum Chi-Squared method (Fig. 5).

More profound rearrangements can be created by intergenic recombinations (both at coding and non-coding sites), which lead to whole gene duplication and deletions. One such rearrangement can be traced in the *L. major *Chr12 *PSA *gene cluster, where *PSA *genes are organised in tandem, tough often separated by one of two unrelated genes of type A or B. The distribution of these intervening genes together with the phylogenic signature of each *PSA *genes suggest that the two pairs of very similar paralogs LmjF12.0810/LmjF12.0940 and LmjF12.0830/LmjF12.0960 were created by a same segmental duplication involving a bloc of five genes (see Additional file 10).

### Positive selection of *PSA *genes

**Figure 6:**
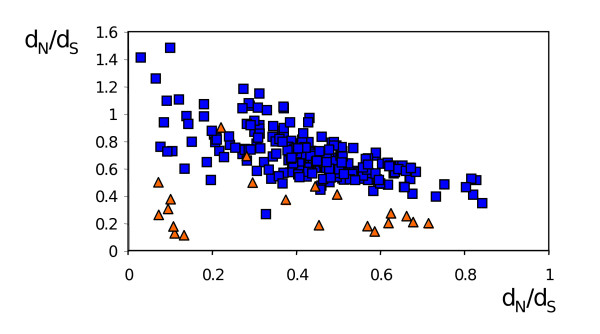
***d*_N_/*d*_S _vs *d*_S _plots for *PSA *genes pairwise comparisons**. DNA alignments covering the LRR domain were generated for each of the Chr5A, B, 9, 12, 21, 31A, B, C *PSA *gene clusters (including genes of all three *Leishmania *species; see Additional file 13 for alignment of Chr12 genes). After reducing very homologous genes (with *d*_S_<0.02) to single entities, *d*_N_/*d*_S _and *d*_S _values were calculated for all pairwise comparions inside a given cluster. Orange triangles: Chr5A, B, 9, 21, 31A, B, C clusters; blue squares: Chr12 cluster. Arrows point to values for *L. major*-*L. infantum *ortholog comparisons.

The high gene diversity found for the Chr12 subfamily in *L. major *and *L. infantum *could be indicative of selective pressure acting on these genes. The preservation of a high number of paralogs on chromosome 12 (with the exception of the single *L. braziliensis *Chr12 gene) in comparison to that of paralogs found on the other chromosomes (one or two copies only) is itself an indication that diversification is taking place in the Chr12 subfamily. This is strengthened by the high protein sequence diversity among the *L. infantum *and *L. major *paralogs of Chr 12. After grouping pairs of genes with *d*_S _values below 0.02 (recently duplicated genes) as single entities, we found that the median pairwise *p*-distances (calculated over the coding sequence deleted of the Thr/Ser and Cys-rich/transmembrane C-terminal domains) for the *L. infantum *and *L. major *Chr12 paralogs were 0.336 and 0.285, respectively (it was only slightly higher, 0.348, for the *L. infantum*-*L. major *genes pairwise comparison). That is to compare to the high protein similarity found between the *L. infantum *and *L. major *orthologs of Chr5, 9, 21, 31, ranging from 91 to 95% identity (median = 94%). Orthologs of *PSA *genes on Chr5, 9, 21, 31 are therefore much more conserved even than paralogs of Chr12. To gain a better insight on selective pressure, we conducted analysis of ratios of *d*_S_, the number of synonymous substitutions per synonymous sites, to *d*_N_, the number of non-synonymous substitutions per non-synonymous sites, over alignments covering the entire LRR domain. We calculated a median *d*_N_/*d*_S _ratio of 0.26 for the pairwise comparison of Chr5, 9, 21, 31 orthologs of *L. infantum *and *L. major *(0.26 when those of *L. braziliensis *where included). The pairwise comparison between all genes of Chr12 of *L. infantum *and *L. major *(after grouping pairs of genes with *d*_S _values below 0.02 as single entities) revealed a much higher median *d*_N_/*d*_S _of 0.74 (0.71 and 0.86 when comparing only paralogs of *L. major *and *L. infantum*, respectively). Fig. 6 shows the *d*_N_/*d*_S _versus *d*_S _plot of all pairwise comparisons. *d*_N_/*d*_S _values of the Chr5,9,21,31 orthologs are quite uniformly distributed along the *d*_S _axis, well below those of the Chr12 genes, with the exception of two points which refer to the same LbrM31_V2.1680 gene. It is worth noting that the *d*_N_/*d*_S _values associated with the lowest *d*_S _values for the Chr12 paralogs (and where the alignments are the most confident) are relatively high. The two ortholog pairs of Chr12 genes (LmjF12.1090/LinJ12_v4.0671 and LmjF12.0850, LmjF12.1070/LinJ12_v4.0663) position somewhat outside the distribution of Chr12 genes towards low *d*_N_/*d*_S _values, especially for LmjF12.1090/LinJ12_v4.0671, whose *d*_N_/*d*_S _value (0.31) rather falls inside the Chr5,9,21,31 ortholog values. These results suggest that the *PSA *genes on chromosome 5, 9, 21 and 31, as well as orthologs LmjF12.1090/LinJ12_v4.0671 and LmjF12.0850, LmjF12.1070/LinJ12_v4.0663 of chromosome 12 have been more subjected to purifying selection than the other Chr12 genes.

**Figure 7:**
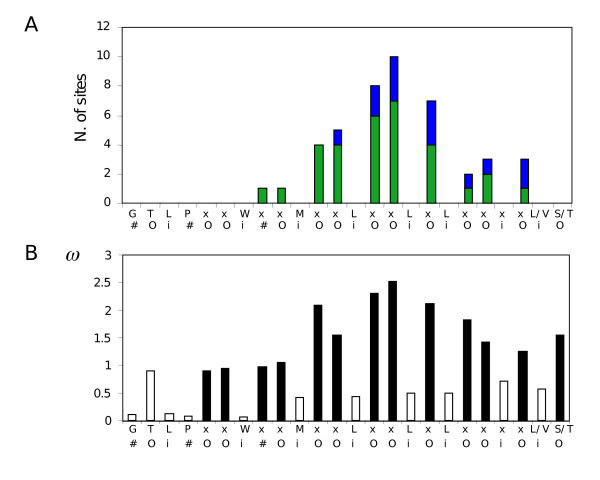
**Distribution of the predicted positively selected sites along the LRR amino acid consensus sequence**. A) A DNA alignment covering the LRR domain plus the flanking N-ter and CGC-C2 domains of all Chr12 *PSA *genes (Additional file 16) was analysed by codeml. A) The predicted positively selected sites were plotted against their positioning in the LRR domain. Green bars represent the number of predicted positive sites with probabilities greater than 95% and blue bars, eventual increments when considering sites with probabilities greater than 90%. o, i and # refer to positions predicted to reside outside, inside or "in between" in the PSA LRR 3D structure. B) Means of *d*_N_/*d*_S _(*ω*) at each residue position in the LRR were calculated from the posterior *d*_N_/*d*_S _values of all sites sharing the same position in the LRR. Filled bars: variable residues; open bars: conserved residues.

A more incisive method to detect positive selection involves analysing *d*_N_/*d*_S _ratios at each codon site. Indeed, *d*_N_/*d*_S _ratios calculated over the entire length of the sequence cannot always identify positive selection (*d*_N_/*d*_S _> 1). That is because only a subset of sites in a given gene is subjected to diversifying selection. The alignment of the entire coding sequence of the Chr12 genes, except the C-terminal Cys-rich and transmembrane domains was analysed using codeml of the PAML package. Likelihood ratio test analysis of three pairs of simple and more complex models of *d*_N_/*d*_S _categories (M1a/M2a, M7/M8 and M8a/M8) all revealed the presence of positive selection with high probabilities (*P *< 0.001). The M1a/M2a models comparison predicted the presence of 43 positively selected sites with *P *< 0.05 (59 sites with *P *< 0.1), among which 33 (44 with *P *< 0.1) were located in the LRR domain. Very similar results were obtained with the M7/M8 and M8a/M8 model comparisons. Codeml also revealed the presence of positive selection (*P *< 0.001) in alignments comprising all Chr 12 genes of either *L. infantum *or *L. major *alone, or the 7 closely related genes of *L. major *12B cluster (see tree of Fig. 3). On the other hand, *PSA *genes on the other chromosomes could not be analysed properly by codeml: each of these phylogenetically defined clusters possess too few genes (2 to 4), and those often offer poor diversity. It is known that inference of positive selection by codeml can lead to false positives when the genes under investigation have a recombination history [22]. To minimise the impact of recombination, we performed codeml analysis on single or contiguous pairs of LRRs of the Chr12 genes. The majority of these analyses revealed the presence of positive selection and could identify sites under positive selection. Under model M8, still 15 such sites were found at P < 0.05 for pairs of LRRs. We then asked if the numerous predicted positively selected sites found above by codeml for the entire LRR domain of Chr12 genes corresponded to particular positions in each LRR. Fig. 7A shows that all these sites are located at non-conserved sites of the LRR consensus sequence. The posterior *d*_N_/*d*_S _values calculated at each site by codeml were grouped according to their corresponding amino acid position in the LRR and a mean *d*_N_/*d*_S _value was determined for each residue position in the LRR (Fig. 7B). As expected, variable positions have higher *d*_N_/*d*_S _values than more conserved positions. An even better correlation is observed if we consider the localisation of the sites in the 3D structure of the LRRs. Crystal structures and computer modelling of several LRR domains in other proteins allow for the prediction of the positioning of residues outside or inside the LRR domain 3D structure [23]. The relatively well conserved hydrophobic residues are involved in structuring of the consecutive α-helices and $\tilde{\beta }$-sheet domains of the LRRs that arrange into a horseshoe shape, and are buried inside the structure. On the opposite, the most divergent residues of the LRR orientate outward. It is clear from Fig. 7A that positively selected sites all correspond to positions predicted to reside at the surface of the LRR domain structure. In addition, the latter positions have higher posterior mean values of *d*_N_/*d*_S _(Fig. 7B).

### 3' Intergenic region

Expression of some *PSA *genes of Chr12 has been shown to be stage specific. Strong increases in the levels of both mRNA and protein were observed during metacyclogenesis *in vitro *[10]. This regulation was shown to depend on the 3' intergenic region (IR) of the *PSA *gene [17]. The question arises, then, as to whether the different classes of *PSA *might be regulated the same way. We therefore wished to compare the phylogeny of the 3' (IR) to that obtained above for the coding sequence. Around 2 kb of the 3' IR of *PSA *genes from all three *Leishmania *species (with few exceptions for *L. infantum *and *L. braziliensis *due to gaps in their sequences) were analysed by local BLAST and aligned by Dialign2. We found that the 3' IRs are conserved between all orthologs (and pseudo-orthologs for he Chr12 clade), but show no significant homology between genes of the 8 different *PSA *clades (05, 09, 12, 21, 31A, B, C). While the similarities found for pairwise comparisons involving *L. braziliensis *sequences are variable and relatively low, those of the other comparisons are high. The *L. infantum*/*L. major *orthologs of clades 5, 9, 21, 31A, B, C are equally well conserved (ranging from 87 to 92% identity; see Additional file 11 for sequence alignments), as are the set of pseudo-orthologs and paralogs of clade 12 (median = 95% identity; see Additional file 12 for sequence alignment). Yet, the alignment of the 3' IR of these Chr12 *PSA *genes is quite mosaic, showing large blocks of deletions/insertions. Phylogenetic analysis of an alignment retaining 1.2 kb of domains conserved in at least 70% of the genes revealed that Chr12 genes cluster according to the species (not shown). This is reminiscent of the extremities of the coding regions (Fig. 2). Functional studies will be necessary to determine if this high degree of conservation is the result of gene conversion or rather, of purifying selection driven by imperatives of gene expression. In any case, if indeed the 3' IR of the different *PSA *clades play a role in the control of gene expression, either the pattern of expression is variable between the clades or the cues governing this control are subtler than the primary sequence per se.

## Discussion and Conclusion

Thanks to the whole genome sequencing of three *Leishmania *species, we could draw the complete picture of the *PSA *gene family. Up to now, only a few genes belonging to the Chr12 subfamily had been characterised. Also, *L. braziliensis *was thought not to contain any gene for that subfamily. We have proposed a nomenclature for all *PSA *genes (see Fig. 2 and 3). We have kept the *PSA *letters, even tough it is now known that these genes are expressed both in promastigote and amastigote stages and code not only for membrane proteins at the surface of the parasite but also for excreted soluble proteins. Follow the chromosome number and other symbols that refer to the phylogenetic *PSA *gene clusters.

PSAs are basically LRR repeats containing proteins, which are either membrane-bound at the surface of *Leishmania *or soluble as excreted proteins. These features suggest that this protein family could be involved in host-parasite interactions. The diversity found in PSAs, which concerns mainly the LRR domain, would be consistent with this role. This diversity is not accounted only by the sequence divergence of the LRR repeats but also by the number of these repeats present in each protein, which varies from 2 to 25 and could influence both the shape and the space occupied by PSAs, and thereby their binding properties. Two other factors of diversity in PSA reside outside the LRR domain. One concerns the Thr/Ser-rich domain, whose length varies considerably and can be totally absent in some PSAs. Although there is some indication that PSAs could be glycosylated [24,25], both the sites of glycosylation and the nature of the amino acid-glycan linkage are unknown. Most of these Thr/Ser-rich domains contain a signature present in *T. cruzi *mucins which is known to be the site of O-glycosylation. Finally, membrane attachment determinants (which result mostly in anchoring through GPI) define two destinations for PSA proteins: excretion or attachment to the parasite's surface. Although the majority are predicted to be membrane bound, PSAs predicted to be secreted are found both as strongly conserved proteins between species (PSA21 in all three species; LiPSA12A and LmPSA12A) and as species specific proteins (LiPSA12I and most LmPSA12C). This suggests that the presence of these C-terminal truncated forms of PSAs is neither fortuitous nor the products of pseudogenes, but that secretion plays a fundamental role in *Leishmania*.

*PSA *genes distribute into eight subfamilies. In view of the found orthologies, the most parsimonious scenario would be that the last common ancestor (LCA) of *L. major*, *L. infantum *and *L. braziliensis *had nine genes: one in each of chromosomes 9, 12 and 21, two on chromosome 5 and three on chromosome 31. Among the *PSA *subfamilies, the Chr12 subfamily is the most diversified one, at least in *L. major *and *L. infantum*. This is brought about by nucleotide substitutions as well as unequal gene recombination, which modulates both the number of LRRs in PSA proteins and the number of genes in the family, and generates chimeric genes. The evolution of the Chr12 subfamily of *PSA *genes is probably best explained by a birth-and-loss process, by which the gene family is either expanded or contracted, while *PSA *genes are individually submitted to diversifying selection. We have detected clear evidence of positive selection for the Chr12 genes in both *L. major *and *L. infantum*, while the existence of orthologs, divergent paralogs, as well as probable pseudogenes (LmjF12.1005, LinJ12_v4.0662) all point to a birth-and-loss model. We note, however, that the extremities of the coding regions and the 3' IR of the Chr12 *PSA *genes are all species specific (no orthologies can be inferred) and remarkably similar (some stretches are even devoid of synonymous substitutions), suggesting that some gene homogenisation might have taken place by gene conversion. In contrast to the Chr12 subfamily, the seven other subfamilies seem to evolve in a more conservative way. Not only all genes of Chr5, 9, 21 and 31 from the three *Leishmania *species cluster as more similar orthologs but evidence of recombination is scarce. One finds only one pair of paralogs (the *L. major *Chr31A subfamily), and the number of LRR repeats is the same for each group of orthologs (except one difference in Chr21 subfamily). Furthermore, diversifying selection apparently does not act on these genes, as evidenced by *d*_N_/*d*_S _values well under those obtained for the Chr12 genes. We would therefore postulate the latter subfamilies to be involved in basic slowly evolving host-parasite interactions, whereas the Chr12 subfamily could be linked to more dynamic adaptative responses like optimisation of cellular infection (internalisation and maintenance in the macrophage) and escape from the immune system. The latter could also be related to the *Leishmania *species identities like the nature of the sand fly species infected and the clinical symptoms. Still, diversification of Chr12 genes apparently does not offer any advantage for *L. braziliensis*. At this stage, it is hard to find out what kind of adaptative role these genes could play in *L. major *and *L. infantum*, which would be dispensable for *L. braziliensis*.

Diversifying selection has been shown to drive evolution of genes involved in host-pathogen interactions. These include virulence genes, as found in *Streptococci *[26] and *Listeria monocytogenes *[27], as well as resistance genes, like the numerous plant *NBS-LRR *genes [28], the primate *Trim5α *[29] genes involved in restriction of retroviruses replication and many gene classes involved in the *Drosophila *innate immune system [30]. Interestingly, the *Listeria *virulence gene *inlA *and the *NBS-LRR *genes possess LRR domains, which were shown to evolve under positive selection. In the present work, we found that in the Chr12 *PSA *gene subfamily of two *Leishmania *species, LRR domains evolve under strong positive selection. Most notably, the several residues submitted to diversifying selection are predicted to reside at the protein's surface. The importance of this evolving LRR protein interface in the parasite's life cycle awaits the determination of the precise PSA biological function and the identification of eventual host PSA interactors.

In conclusion, we have identified eight PSA subfamilies. The phylogenetic distance separating them, which is paralleled by the divergence of their LRR domains, appears large enough to reflect functional specificities. The strong conservation of the orthologs of the subfamilies 5A, 5B, 9, 21, 31A, B and C suggest that they play essential roles. Generation of knock outs and gene expression analysis of all individual *PSA *genes will be needed to explore the importance of these genes in the different stages of the life cycle of *Leishmania*, and eventually in adaptation of the parasites to their specific insect or mammalian host. In comparison to the seven subfamilies above, the PSA12 subfamily is evolving much more rapidly, (at least in *L. major *and *L. infantum*) as revealed by the high number of paralogs and the presence of strong diversifying selection. Sequencing of these genes in several strains of *L. major *and *L. infantum *is expected to show a high incidence of polymorphism.

## Methods

### Alignment of PSA amino acid and nucleotide sequences and identification of membrane anchorage determinants

PSA protein and DNA sequences were extracted from the *L. major*, *L. infantum *and *L. braziliensis *database http://www.genedb.org/ after BLAST analysis. The latest versions of the genome sequences were used, i.e. 5.2 for *L. major*, 2.0 for *L. braziliensis *and 3.0 (and 4.0 for chromosome 12) for *L. infantum*. Protein sequences were aligned with ClustalX 2.0 [31] followed by manual correction. These alignments were transposed to the corresponding genomic DNA alignments by protal2dna http://mobyle.pasteur.fr/cgi-bin/MobylePortal/portal.py?form=tranalign. The presence of signal peptide and transmembrane domains was reported as found in annotations of the *Leishmania *genomes http://www.genedb.org/. The prediction of GPI-anchorage was performed by GPI-SOM http://gpi.unibe.ch/[32]. Genomic sequences flanking the *PSA *ORFs in 3' were aligned with Dialign 2.2.1 [33].

### Phylogenetic analysis

Phylogenetic trees of Fig. 2 and 3 were obtained from nucleotide sequence alignments by maximum likelihood (PhyML v2.4.4) [34]. The selection of the nucleotide substitution model (and eventually of amino acids substitution) was determined by ModelGenerator http://distributed.cs.nuim.ie/multiphyl.php[35]. The HKY+I+G (Fig. 2) and TN93 +G+I (Fig. 3) nucleotide models were used, with the base frequency, Ts/Tv ratio, and gamma distribution parameter estimated by PhyML. These trees were compared to those obtained by PhyML from the corresponding amino acid sequences (WAG+G model of amino acids substitution) or with a distance method implemented in MEGA v3.1 [36] using both DNA (TN93) or amino acids (JTT model) alignments. Occasionally (see text in Results section), a codon model (*d*_S_, number of synonymous substitutions per synonymous sites; Nei-Gojobori method, JC model) as implemented in MEGA was used to calculate distance matrix. Trees were annotated and visualised by Treedyn (v194.3) [37].

### Positive selection

One level of positive selection analysis involved calculation of *d*_N_/*d*_S _ratios between pairs of sequences (MEGA, Nei-Gojobori method, JC model). *ω *= *d*_N_/*d*_S _< 1 corresponds to purifying (negative) selection, *ω *= 1 to neutral evolution (absence of selection), and *ω *> 1 indicating adaptive evolution (positive selection). The maximum likelihood based method implemented in the codeml program of the PAML package (v3.15) [38] was used to detect positive selection as well as identifying positively selected sites, when the number of related sequences was sufficiently high. To detect positive selection, we performed likelihood ratio test (LRT) analysis between three pairs of models to determine whether particular models provided a significantly better fit to the data: M1a ("Nearly-Neutral" model) vs. M2a ("PositiveSelection" model), M7 ("beta" model) vs. M8 ("beta&ω" model) and M8a vs. M8. Only models M2a and M8 allow for site categories evolving under positive selection (see Yang et al. 2005 [39] for further details on the models). Positive selection is inferred when the (2Δλ) statistic is greater than critical values 1) of the Chi square distribution for a degree of freedom of 2 (M1a/M2a. and M7/M8 comparisons) or 2) of a 50:50 mixture of point mass 0 and Chi square (critical values are 2.71 at 5% and 5.41 at 1%; M8a/M8 comparison). Only predicted positively selected sites with posterior BEB (Bayes empirical Bayes) probabilities greater than 0.95 or 0.90 were retained.

## Authors' contributions

AD carried out the data mining, the sequence alignments, bioinformatic analysis and wrote the manuscript. ALB conceived the study and participated in writing the manuscript. All authors read and approved the final manuscript.

## Supplementary Material

Additional file 1 **DNA sequence alignment of all *PSA *genes**.T/S- rich, TM/GPI anchor and LRR domains (except the most C-terminal LRR repeat) were excluded from the alignment, as well as a unique repeat of the LRR-like domain in LinJ12_v4.0670. Click here for file

Additional file 2 Amino acid sequence alignment of the LRR domain of proteins of cluster 5A. Click here for file

Additional file 3 Amino acid sequence alignment of the LRR domain of proteins of cluster 5B. Click here for file

Additional file 4 Amino acid sequence alignment of the LRR domain of proteins of cluster 9. Click here for file

Additional file 5 Amino acid sequence alignment of the LRR domain of proteins of cluster 21. Click here for file

Additional file 6 Amino acid sequence alignment of the LRR domain of proteins of cluster 31A. Click here for file

Additional file 7 Amino acid sequence alignment of the LRR domain of proteins of cluster 31B. Click here for file

Additional file 8 Amino acid sequence alignment of the LRR domain of proteins of cluster 31C. Click here for file

Additional file 9 **Amino acid sequence alignment of the LRR domain of all PSA proteins of Chr12**. Some repeats unique to certain proteins were deleted. Click here for file

Additional file 10 **Structure of the *PSA *gene cluster on chromosome 12 of *L. major***. The indicated LmjF12.xxxx *PSA *genes (rectangular boxes) and the intervening type A (LmjF12.0880, 0885, 0905, 0995, 1015) and B (LmjF12.0715, 0860, 0800, 0820, 0840, 0880, 0895, 0930, 0950, 0970, 1030, 1050) paralogs form a single uninterrupted cluster. *PSA *genes belonging to the same phylogenetic sub-clusters are represented by the same colour. Arrows link highly similar genes that probably arose by duplication of a cluster of five genes. Click here for file

Additional file 11 **Alignment of 3' intergenic region of *PSA *genes from clades 5A, B, 9, 21, 31A, B, C**. Sequences are listed according to their clade in the above order. Alignments were performed only between orthologs. 3' IR region of gene LbrM31_V2.1670 is not determined in the genome database. Click here for file

Additional file 12 Alignment of 3' intergenic region of *PSA *genes from clade 12 (*L. infantum *and *L. major*). Click here for file

Additional file 13 **DNA sequence alignment of the LRR domain of all *PSA *genes of Chr12**. DNA alignment transformed from that of Additional file 9 and used to infer phylogenetic tree of Fig. 3. Click here for file

Additional file 14 **DNA sequence alignment of a subset of Chr12 *PSA *genes used for recombination analysis of gene LmjF12.0990 in Fig.**5. Click here for file

Additional file 15 **DNA sequence alignment of a subset of Chr12 *PSA *genes used for recombination analysis of gene LmjF12.0960 in Fig.**5. Click here for file

Additional file 16 **DNA sequence alignment of Chr12 *PSA *genes used for positive selection analysis in Fig.**7. Click here for file

## References

[B1] Schlein Y *Leishmania *and Sandflies: interactions in the life cycle and transmission Parasitol Today 1993 9 7 255 258 15463772 10.1016/0169-4758(93)90070-v

[B2] Banuls AL Hide M Prugnolle F *Leishmania *and the leishmaniases: a parasite genetic update and advances in taxonomy, epidemiology and pathogenicity in humans Adv Parasitol 2007 64 1 109 17499100 10.1016/S0065-308X(06)64001-3

[B3] Frommel TO Button LL Fujikura Y McMaster WR The major surface glycoprotein (GP63) is present in both life stages of *Leishmania* Mol Biochem Parasitol 1990 38 1 25 32 2181303 10.1016/0166-6851(90)90201-v

[B4] Jimenez-Ruiz A Boceta C Bonay P Requena JM Alonso C Cloning, sequencing, and expression of the PSA genes from *Leishmania infantum* Eur J Biochem 1998 251 1–2 389 397 9492309 10.1046/j.1432-1327.1998.2510389.x

[B5] Ilg T Proteophosphoglycans of *Leishmania* Parasitol Today 2000 16 11 489 497 11063860 10.1016/s0169-4758(00)01791-9

[B6] Rittig MG Bogdan C *Leishmania*-host-cell interaction: complexities and alternative views Parasitol Today 2000 16 7 292 297 10858648 10.1016/s0169-4758(00)01692-6

[B7] Santarem N Silvestre R Tavares J Silva M Cabral S Maciel J Cordeiro-da-Silva A Immune response regulation by leishmania secreted and nonsecreted antigens J Biomed Biotechnol 2007 2007 6 85154 17710243 10.1155/2007/85154 PMC1940321

[B8] Olivier M Gregory DJ Forget G Subversion mechanisms by which *Leishmania *parasites can escape the host immune response: a signaling point of view Clin Microbiol Rev 2005 18 2 293 305 15831826 10.1128/CMR.18.2.293-305.2005 PMC1082797

[B9] Handman E Osborn AH Symons F van Driel R Cappai R The *Leishmania *promastigote surface antigen 2 complex is differentially expressed during the parasite life cycle Mol Biochem Parasitol 1995 74 2 189 200 8719160 10.1016/0166-6851(95)02500-6

[B10] Beetham JK Donelson JE Dahlin RR Surface glycoprotein PSA (GP46) expression during short- and long-term culture of *Leishmania chagasi* Mol Biochem Parasitol 2003 131 2 109 117 14511809 10.1016/s0166-6851(03)00197-x

[B11] Lincoln LM Ozaki M Donelson JE Beetham JK Genetic complementation of *Leishmania *deficient in PSA (GP46) restores their resistance to lysis by complement Mol Biochem Parasitol 2004 137 1 185 189 15279966 10.1016/j.molbiopara.2004.05.004

[B12] Pizarro-Cerda J Cossart P Bacterial adhesion and entry into host cells Cell 2006 124 4 715 727 16497583 10.1016/j.cell.2006.02.012

[B13] Seepersaud R Hanniffy SB Mayne P Sizer P Le Page R Wells JM Characterization of a novel leucine-rich repeat protein antigen from group B *streptococci *that elicits protective immunity Infect Immun 2005 73 3 1671 1683 15731068 10.1128/IAI.73.3.1671-1683.2005 PMC1064916

[B14] Athman R Philpott D Innate immunity via Toll-like receptors and Nod proteins Curr Opin Microbiol 2004 7 1 25 32 15036136 10.1016/j.mib.2003.12.013

[B15] McHale L Tan X Koehl P Michelmore RW Plant NBS-LRR proteins: adaptable guards Genome Biol 2006 7 4 212 16677430 10.1186/gb-2006-7-4-212 PMC1557992

[B16] Lohman KL Langer PJ McMahon-Pratt D Molecular cloning and characterization of the immunologically protective surface glycoprotein GP46/M-2 of *Leishmania amazonensis* Proc Natl Acad Sci USA 1990 87 21 8393 8397 2236047 10.1073/pnas.87.21.8393 PMC54962

[B17] Myung KS Beetham JK Wilson ME Donelson JE Comparison of the post-transcriptional regulation of the mRNAs for the surface proteins PSA (GP46) and MSP (GP63) of *Leishmania chagasi* J Biol Chem 2002 277 19 16489 16497 11856749 10.1074/jbc.M200174200

[B18] Peacock CS Seeger K Harris D Murphy L Ruiz JC Quail MA Peters N Adlem E Tivey A Aslett M Comparative genomic analysis of three *Leishmania *species that cause diverse human disease Nat Genet 2007 39 7 839 847 17572675 10.1038/ng2053 PMC2592530

[B19] Ivens AC Peacock CS Worthey EA Murphy L Aggarwal G Berriman M Sisk E Rajandream MA Adlem E Aert R The genome of the kinetoplastid parasite, *Leishmania major* Science 2005 309 5733 436 442 16020728 10.1126/science.1112680 PMC1470643

[B20] Kajava AV Structural diversity of leucine-rich repeat proteins J Mol Biol 1998 277 3 519 527 9533877 10.1006/jmbi.1998.1643

[B21] Lole KS Bollinger RC Paranjape RS Gadkari D Kulkarni SS Novak NG Ingersoll R Sheppard HW Ray SC Full-length human immunodeficiency virus type 1 genomes from subtype C-infected seroconverters in India, with evidence of intersubtype recombination J Virol 1999 73 1 152 160 9847317 10.1128/jvi.73.1.152-160.1999 PMC103818

[B22] Anisimova M Nielsen R Yang Z Effect of recombination on the accuracy of the likelihood method for detecting positive selection at amino acid sites Genetics 2003 164 3 1229 1236 12871927 10.1093/genetics/164.3.1229 PMC1462615

[B23] Kajava AV Kobe B Assessment of the ability to model proteins with leucine-rich repeats in light of the latest structural information Protein Sci 2002 11 5 1082 1090 11967365 10.1110/ps.4010102 PMC2373561

[B24] Kahl LP McMahon-Pratt D Structural and antigenic characterization of a species- and promastigote-specific *Leishmania *mexicana amazonensis membrane protein J Immunol 1987 138 5 1587 1595 3543130

[B25] Murray PJ Spithill TW Handman E The PSA-2 glycoprotein complex of *Leishmania major *is a glycosylphosphatidylinositol-linked promastigote surface antigen J Immunol 1989 143 12 4221 4226 2592773

[B26] Anisimova M Bielawski J Dunn K Yang Z Phylogenomic analysis of natural selection pressure in *Streptococcus *genomes BMC Evol Biol 2007 7 154 17760998 10.1186/1471-2148-7-154 PMC2031904

[B27] Orsi RH Ripoll DR Yeung M Nightingale KK Wiedmann M Recombination and positive selection contribute to evolution of *Listeria monocytogenes inlA* Microbiology 2007 153 Pt 8 2666 2678 17660431 10.1099/mic.0.2007/007310-0

[B28] Lehmann P Structure and evolution of plant disease resistance genes J Appl Genet 2002 43 4 403 414 12441626

[B29] Sawyer SL Wu LI Emerman M Malik HS Positive selection of primate TRIM5alpha identifies a critical species-specific retroviral restriction domain Proc Natl Acad Sci USA 2005 102 8 2832 2837 15689398 10.1073/pnas.0409853102 PMC549489

[B30] Sackton TB Lazzaro BP Schlenke TA Evans JD Hultmark D Clark AG Dynamic evolution of the innate immune system in *Drosophila* Nat Genet 2007 39 12 1461 1468 17987029 10.1038/ng.2007.60

[B31] Thompson JD Gibson TJ Plewniak F Jeanmougin F Higgins DG The CLUSTAL_X windows interface: flexible strategies for multiple sequence alignment aided by quality analysis tools Nucleic Acids Res 1997 25 24 4876 4882 9396791 10.1093/nar/25.24.4876 PMC147148

[B32] Fankhauser N Maser P Identification of GPI anchor attachment signals by a Kohonen self-organizing map Bioinformatics 2005 21 9 1846 1852 15691858 10.1093/bioinformatics/bti299

[B33] Morgenstern B DIALIGN 2: improvement of the segment-to-segment approach to multiple sequence alignment Bioinformatics 1999 15 3 211 218 10222408 10.1093/bioinformatics/15.3.211

[B34] Guindon S Gascuel O A simple, fast, and accurate algorithm to estimate large phylogenies by maximum likelihood Syst Biol 2003 52 5 696 704 14530136 10.1080/10635150390235520

[B35] Keane TM Creevey CJ Pentony MM Naughton TJ McLnerney JO Assessment of methods for amino acid matrix selection and their use on empirical data shows that ad hoc assumptions for choice of matrix are not justified BMC Evol Biol 2006 6 29 16563161 10.1186/1471-2148-6-29 PMC1435933

[B36] Kumar S Tamura K Nei M MEGA3: Integrated software for Molecular Evolutionary Genetics Analysis and sequence alignment Brief Bioinform 2004 5 2 150 163 15260895 10.1093/bib/5.2.150

[B37] Chevenet F Brun C Banuls AL Jacq B Christen R TreeDyn: towards dynamic graphics and annotations for analyses of trees BMC Bioinformatics 2006 7 439 17032440 10.1186/1471-2105-7-439 PMC1615880

[B38] Yang Z PAML: a program package for phylogenetic analysis by maximum likelihood Comput Appl Biosci 1997 13 5 555 556 9367129 10.1093/bioinformatics/13.5.555

[B39] Yang Z Wong WS Nielsen R Bayes empirical bayes inference of amino acid sites under positive selection Mol Biol Evol 2005 22 4 1107 1118 15689528 10.1093/molbev/msi097

