# Supplementary figures and images for: The promastigote surface antigen gene family of the Leishmania parasite: differential evolution by positive selection and recombination
Source: BMC Evol Biol. 2008 Oct 24;8:292. doi: 10.1186/1471-2148-8-292 (PMC2584048; doi:10.1186/1471-2148-8-292)

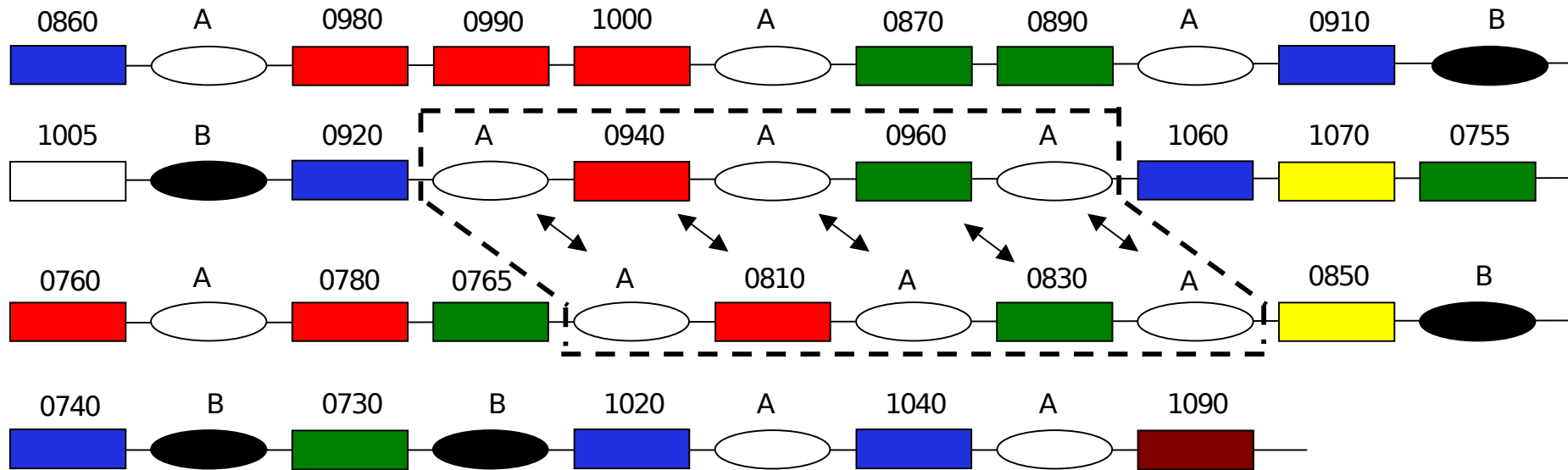

Supplement: Additional file 10 — Structure of the PSA gene cluster on chromosome 12 of L. major. The indicated LmjF12.xxxx PSA genes (rectangular boxes) and the intervening type A (LmjF12.0880, 0885, 0905, 0995, 1015) and B (LmjF12.0715, 0860, 0800, 0820, 0840, 0880, 0895, 0930, 0950, 0970, 1030, 1050) paralogs form a single uninterrupted cluster. PSA genes belonging to the same phylogenetic sub-clusters are represented by the same colour. Arrows link highly similar genes that probably arose by duplication of a cluster of five genes. [file 1471-2148-8-292-S10.pdf]
